# Supplementary material for: Production of a Dulaglutide Analogue by Apoptosis-Resistant Chinese Hamster Ovary Cells in a 3-Week Fed-Batch Process
Source: Pharmaceuticals (Basel). 2025 Dec 16;18(12):1896. doi: 10.3390/ph18121896 (PMC12736101; doi:10.3390/ph18121896)
Supplement: Supplementary file 1 [file pharmaceuticals-18-01896-s001.zip › pharmaceuticals-4024255-supplementary.pdf]

Production of a Dulaglutide Biosimilar by the Apoptosis-Resistant CHO Cells in the 3-Weeks Fed-Batch Process

Supporting Materials

Table S1. Oligonucleotides used.

Name	Sequence 5`-3`
M13-dir	GTTGTAAACGACGGCCAGTG
M13-rev	AGCGGATAACAATTTCACACAGGA
SP6	GATTTAGGTGACACTATAG
T7prom	TAATACGACTCACTATAGGG
SQ-5CH6-F	GCCGCTGCTTCCTGTGAC
IRESArev	AGGTTTCCGGGCCCTCACATTG
AD-DHFR-F	AAGATCTGCCACCATGGTTC
AD-DHFR-R	ATCTAGATTAGTCTTTCTTCTCGTAGAC
AD-GS-BglNcoF	AGATCTGCCACCATGGCCACCTCAGCAAGT
AD-GS-XbaR	TCTAGATTAGTTCTTGTATTGGAAGG
SQ-DUL-F	CGT GTT CTC CTG CTC CG
SQ-DUL-R	CGGAGCAGGAGAACACG

Table S2. List of tryptic peptides of dulaglutide according to MS data.

Range	Sequence	Modification	m/z meas., Da	MW meas., Da	Δ m/z	Peak Retention Time, min	Ion Charge
1 - 20	-.HGEGTFTSDVSSYLEEQAAK.E		539.7509	2154.9746	1.86	17.88	4
1 - 20	-.HGEGTFTSDVSSYLEEQAAK.E		719.3315	2154.9725	0.92	17.88	3
1 - 20	-.HGEGTFTSDVSSYLEEQAAK.E		1078.4925	2154.9704	-0.07	17.88	2
1 - 28	-.HGEGTFTSDVSSYLEEQAAKEFI-AWLVK.G		786.3911	3141.5352	1.8	30.04	4
1 - 28	-.HGEGTFTSDVSSYLEEQAAKEFI-AWLVK.G		1048.1845	3141.5318	0.73	29.99	3
21 - 28	K.EFIAWLVK.G		503.2927	1004.5709	1.39	21.31	2
29 - 50	K.GGGGGGGSGGGGSGGGG-SAESK.Y		775.8267	1549.6389	2.34	1.14	2
51 - 77	K.YGPPCPPCPAPEAAGGPSVFLF-PPKPK.D	Carbamidomethyl: 5, 8	709.607	2834.3989	2.3	19.76	4
51 - 77	K.YGPPCPPCPAPEAAGGPSVFLF-PPKPK.D	Carbamidomethyl: 5, 8	945.8064	2834.3973	1.73	19.76	3
51 - 77	K.YGPPCPPCPAPEAAGGPSVFLF-PPKPK.D	Carbamidomethyl: 5, 8	1418.2038	2834.393	0.19	19.76	2
51 - 84	K.YGPPCPPCPAPEAAGGPSVFLF-PPKPKDTLMISR.T	Carbamidomethyl: 5, 8	913.7104	3650.8126	1.05	20.75	4
78 - 84	K.DTLMISR.T		835.4357	834.4285	1.81	10.85	1
85 - 117	R.TPEVTCVVVDVSQEDPEVQFNWY VDGVEVHNAK.T	Carbamidomethyl: 6	947.9495	3787.769	0.37	23.23	4
85 - 117	R.TPEVTCVVVDVSQEDPEVQFNWY VDGVEVHNAK.T	Carbamidomethyl: 6	1263.5964	3787.7673	-0.08	23.23	3
118 - 130	K.TKPREEQFNSTYR.V	dHex(1)Hex(3)HexNAc(4): 9	775.8437	3099.3458	1.81	6.3	4
118 - 130	K.TKPREEQFNSTYR.V	dHex(1)Hex(4)HexNAc(4): 9	816.3573	3261.4003	2.22	6.22	4
118 - 130	K.TKPREEQFNSTYR.V	dHex(1)Hex(3)HexNAc(4): 9	1034.1224	3099.3454	1.69	6.3	3

118 - 130	K.TKPREEQFNSTYR.V	dHex(1)Hex(4)HexNAc(4): 9	1088.1406	3261.4	2.14	6.24	3
122 - 130	R.EEQFNSTYR.V	dHex(1)Hex(3)HexNAc(4): 5	1309.532	2617.0494	2.19	7.78	2
122 - 130	R.EEQFNSTYR.V	dHex(1)Hex(4)HexNAc(4): 5	1390.5588	2779.103	2.33	7.62	2
131 - 146	R.VVSVLTVLHQDWLNGK.E		603.3413	1807.002	1.55	23.12	3
131 - 146	R.VVSVLTVLHQDWLNGK.E		904.5076	1807.0006	0.76	23.12	2
131 - 149	R.VVSVLTVLHQDWLNGKEYK.C		557.8082	2227.2037	1.62	21.67	4
131 - 149	R.VVSVLTVLHQDWLNGKEYK.C		743.4077	2227.2012	0.5	21.67	3
131 - 149	R.VVSVLTVLHQDWLNGKEYK.C		1114.6056	2227.1967	-1.54	21.67	2
152 - 163	K.VSNKGLPSSIEK.T		629.8557	1257.6969	3.2	8.92	2
168 - 173	K.AKGQPR.E		656.385	655.3777	1.72	1.14	1
168 - 189	K.AKGQPREPQVYTLPPSQEEMTK.N		838.7611	2513.2615	1.23	11.78	3
170 - 173	K.GQPR.E		457.2532	456.2459	3.15	1.14	1
170 - 189	K.GQPREPQVYTLPPSQEEMTK.N		772.385	2314.1332	2.95	12.88	3
170 - 199	K.GQPREPQVYTLPP-SQEEMTKNQVSLTCLVK.G	Carbamidome-thyl: 27	865.1952	3456.7515	3.86	18.36	4
174 - 189	R.EPQVYTLPPSQEEMTK.N		938.9555	1875.8965	2.17	13.67	2
174 - 199	R.EPQVYTLPP-SQEEMTKNQVSLTCLVK.G	Carbamidome-thyl: 23	1007.1789	3018.5149	3.53	19.43	3
190 - 199	K.NQVSLTCLVK.G	Carbamidome-thyl: 7	581.3195	1160.6244	1.8	15.21	2
190 - 199	K.NQVSLTCLVK.G	Carbamidome-thyl: 7	1161.6313	1160.624	1.41	15.21	1
200 - 221	K.GFYPSDIAVEWESNGQPENNYK.T		848.7163	2543.1272	1.22	19.98	3
200 - 221	K.GFYPSDIAVEWESNGQPENNYK.T		1272.5701	2543.1256	0.6	19.98	2
200 - 238	K.GFYPSDIAVEWESNGQPENNYKTTPPVLDSDGS-FFLYSR.L		1476,3533	4426.0382	0.89	24.58	3
222 - 238	K.TTPPVLDSDGSFFLYSR.L		634.6487	1900.9242	1.84	21.11	3
222 - 238	K.TTPPVLDSDGSFFLYSR.L		951.4683	1900.922	0.69	21.11	2
239 - 243	R.LTVDK.S		575.3418	574.3345	3.27	2.6	1
244 - 268	K.SRWQEGNVFSCSVM-HEALHNHYTQK.S	Carbamidome-thyl: 11	609.8841	3044.3843	2.39	15.46	5
246 - 268	R.WQEGNVFSCSVM-HEALHNHYTQK.S	Carbamidome-thyl: 9	561.257	2801.2488	1.75	16.24	5
246 - 268	R.WQEGNVFSCSVM-HEALHNHYTQK.S	Carbamidome-thyl: 9	701.3189	2801.2465	0.93	16.24	4
246 - 268	R.WQEGNVFSCSVM-HEALHNHYTQK.S	Carbamidome-thyl: 9	934.7552	2801.2437	-0.04	16.24	3

Note: Coverage was 95.4%. The arrangement of the peptides on the chromatogram is shown in Figure S1. Glycopeptide 118-130 was detected only in the glycosylated form; the attached glycans were dHex(1)Hex(3)HexNAc(4) and dHex(1)Hex(4)HexNAc(4), i.e., the most common types of biantennary N-glycans, G0F and G1F.

Table S3. Free amino acids in the spent culture broth, basal culture medium, and feeds.

AA name	AA Concentration, mM			Final AA Concentration, mM		Consumed, %		Final AA Concentration vs. Concentration in the EmCD CHO 101 Basal Medium	
	EmCD CHO 101 Basal Medium	101 Feeds	All Input*	#73	#105	#73	#105	#73	#105
Asp	9.15	5.29	93.7	24.65	32.64	74%	65%	270%	357%
Thr	2.07	2.14	36.4	10.96	16.47	70%	55%	530%	796%
Gln	0.00	0.70	11.1	3.26	0.00	71%	100%	n/a	n/a
Ser	4.17	2.03	36.6	4.42	4.18	88%	89%	106%	100%

Glu	0.68	0.74	12.6	12.53	15.76	0%	-25%	1856%	2333%
Pro	1.94	0.88	16.0	5.65	6.92	65%	57%	291%	356%
Gly	1.57	0.00	1.6	3.05	4.33	-94%	-176%	194%	276%
Ala	0.81	0.00	0.8	0.48	1.03	41%	-27%	59%	127%
Cys	0.37	0.39	6.7	1.06	1.94	84%	71%	286%	523%
Val	4.50	1.92	35.2	9.71	13.57	72%	61%	216%	301%
Met	1.43	0.44	8.4	2.24	3.15	73%	63%	157%	220%
Ile	1.87	1.40	24.3	6.40	9.01	74%	63%	342%	481%
Leu	2.94	2.80	47.7	11.34	14.82	76%	69%	386%	505%
Tyr	1.41	0.62	11.3	2.21	2.60	80%	77%	156%	184%
Phe	1.07	1.09	18.5	4.93	6.69	73%	64%	461%	626%
His	0.55	0.37	6.4	1.45	2.15	77%	66%	264%	390%
Lys	4.23	0.92	19.0	6.85	5.94	64%	69%	162%	140%
Arg	2.06	1.07	19.1	3.16	7.03	83%	63%	153%	341%

Notes: * - 16 feeds were introduced during the fed-batch culture. Amino acid concentrations in the EmCD CHO 101 Feed A and Feed B were measured after the dilution in the PBS to the working concentrations (3%+0,3%). Large increase in residual amino acid concentration is marked by orange fill.

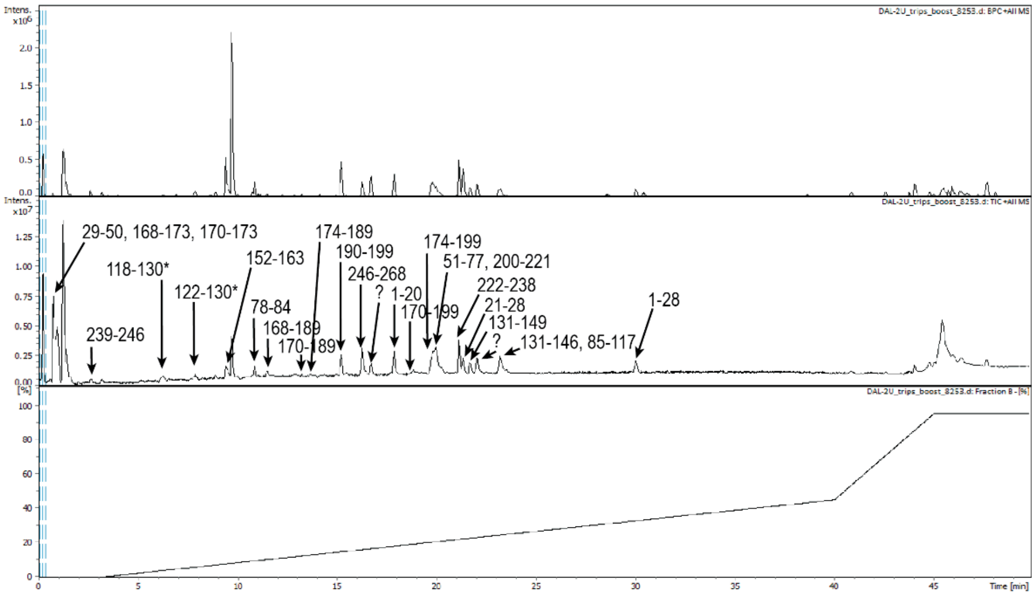

Figure S1. Chromatogram of the separation of a set of dulaglutide tryptic peptides. Arrows and numbers indicate peptides contained in visible peaks of the chromatogram; *—glycopeptide; ?—unidentified peptide.

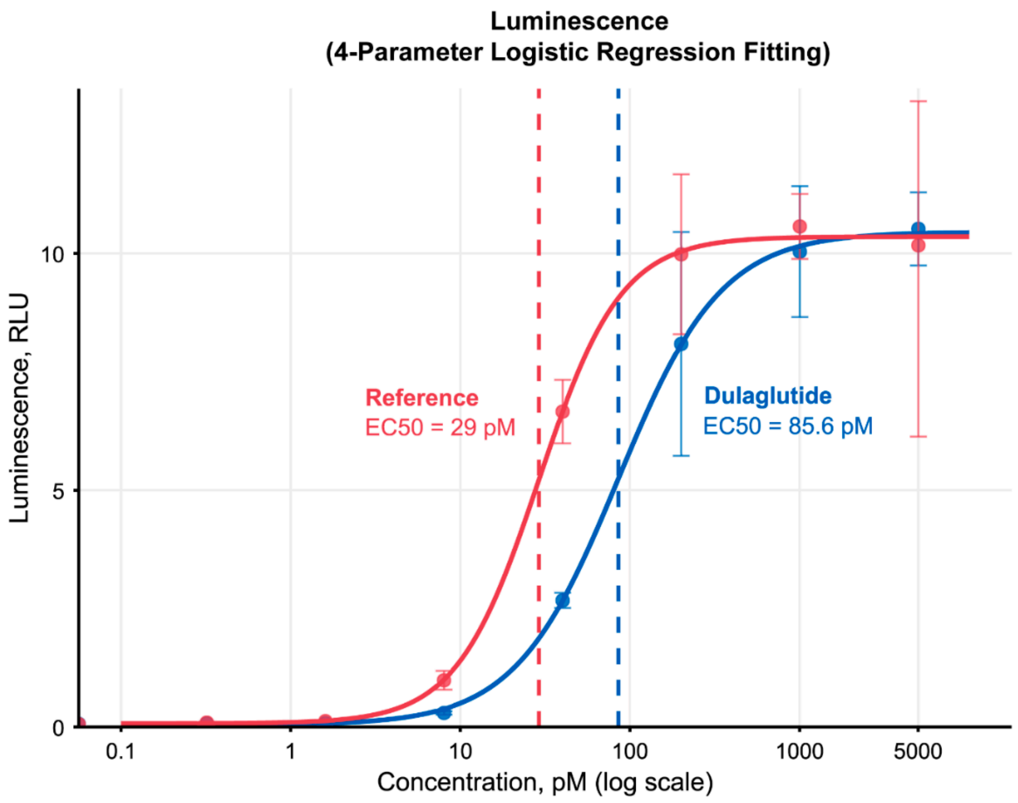

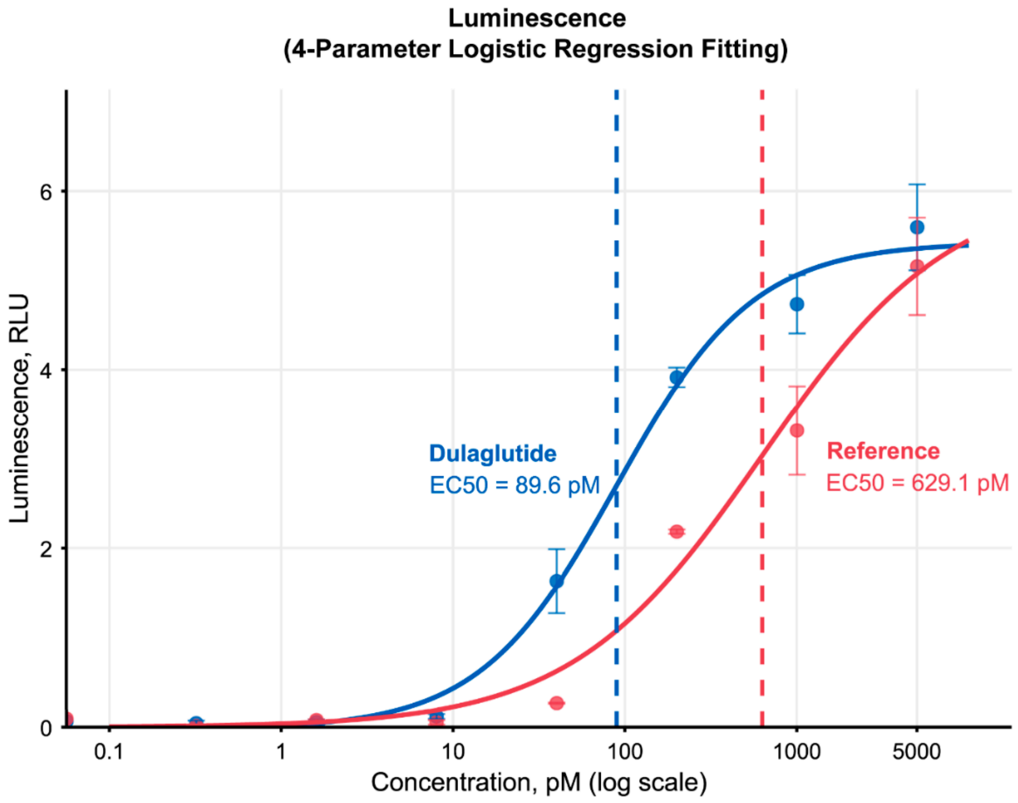

Figure S2. Dulaglutide bioactivity measured in vitro using GLP-1R-expressing HEK293 cell line in comparison with reference original product (Trulicity). Additional experiments. Luminescence signal increase (RLU) under varying agonist concentration.
